# Saliency-Guided Detection of Unknown Objects in RGB-D Indoor Scenes

**DOI:** 10.3390/s150921054

**Published:** 2015-08-27

**Authors:** Jiatong Bao, Yunyi Jia, Yu Cheng, Ning Xi

**Affiliations:** 1 Department of Hydraulic, Energy and Power Engineering, Yangzhou University, Yangzhou 225000, China; 2 Department of Electrical and Computer Engineering, Michigan State University, East Lansing, MI 48824, USA; E-Mails: jiayunyi@msu.edu (Y.J.); chengyu9@msu.edu (Y.C.); xin@msu.edu (N.X.)

**Keywords:** unknown object detection, saliency detection, RGB-D object segmentation

## Abstract

This paper studies the problem of detecting unknown objects within indoor environments in an active and natural manner. The visual saliency scheme utilizing both color and depth cues is proposed to arouse the interests of the machine system for detecting unknown objects at salient positions in a 3D scene. The 3D points at the salient positions are selected as seed points for generating object hypotheses using the 3D shape. We perform multi-class labeling on a Markov random field (MRF) over the voxels of the 3D scene, combining cues from object hypotheses and 3D shape. The results from MRF are further refined by merging the labeled objects, which are spatially connected and have high correlation between color histograms. Quantitative and qualitative evaluations on two benchmark RGB-D datasets illustrate the advantages of the proposed method. The experiments of object detection and manipulation performed on a mobile manipulator validate its effectiveness and practicability in robotic applications.

## 1. Introduction

**Figure 1:**
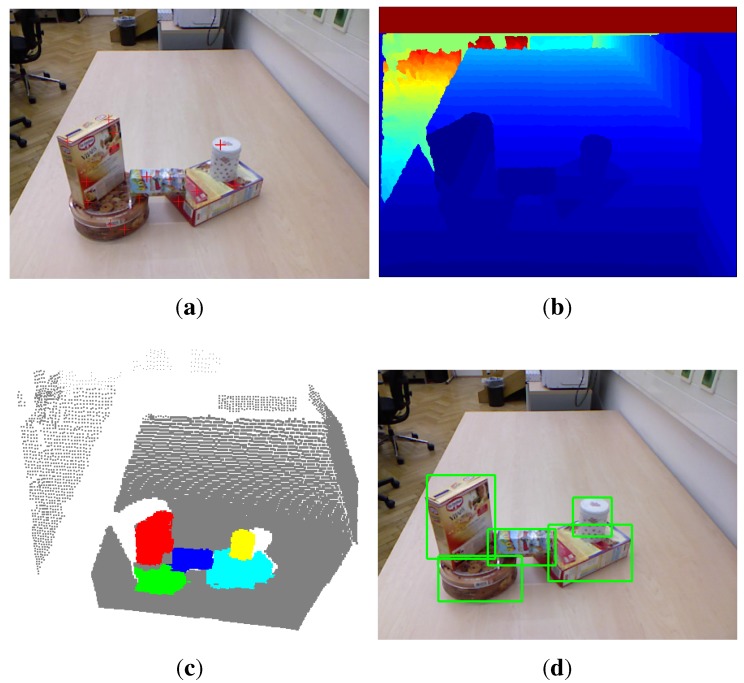
Given an RGB image (**a**) along with per-pixel depth information, (**b**) captured by an RGB-D camera, we detect possible positions (denoted by red plus) of previously unseen objects in a biologically-inspired way, generate and model object hypotheses, segment RGB-D objects in a multi-class labeling framework and, finally, refine the detected objects. The object detection result for the input scene is shown as a colored point cloud (**c**), where the background has gray color, while the detected objects have other colors, respectively. (**d**) Shows the detected objects with bounding boxes.

When situated in an unfamiliar visual environment, humans become so natural at rapidly focusing, segmenting and recognizing objects, so as to help understand their situations, facilitate mobility and interact with objects. However, this remains an ongoing challenge for intelligent machine systems. Many research communities have been dedicated to the core problem of object detection, focusing on segmenting out not only pre-learned objects [1,2], but also previously unknown objects [3,4,5,6,7] from various distractors in the environments. In contrast to known objects, the detection of previously unknown objects is more challenging, since it cannot rely on preexisting object models. The ability to detect unknown objects becomes much more crucial, especially in robotic applications, which need to act on new objects in the environments. This work focuses on the problem of segmenting previously unknown objects. Therefore, we will not employ any prior knowledge about the objects to be detected. We begin by capturing pairs of RGB and depth images, as shown in Figure 1a,b, from a Kinect RGB-D camera. Because areas that absorb or scatter the Kinect IR are filled with a zero pixel value, the depth image is smoothed by replacing the zero value pixels with the statistical mode of the surrounding 25 pixels [8]. Meanwhile, the point cloud of the scene is reconstructed and voxelized. We first propose a new color-based saliency detection method to detect salient positions in the RGB image and employ a depth saliency method to find more salient positions that have high center-surround depth contrast in the depth image. The two types of salient positions (e.g., denoted as a red plus in Figure 1a) are both projected to the 3D scene and serve as candidate seed points of object hypotheses. We then model the object hypotheses using the 3D shape (e.g., object size) efficiently and perform object inference in a computational framework of multi-class 3D scene labeling. The labeling results are further refined by merging the labeled objects that are spatially connected and with high correlation between color histograms. Figure 1c shows the final labeling result for the example scene where different detected objects are colored respectively. Figure 1d shows the corresponding bounding boxes.

The contribution of this paper is three-fold: (i) we propose a new color-based saliency detection method, which is enhanced by a depth saliency method to find sufficient salient positions that are similar to the human eye fixations; (ii) we employ 3D shape to efficiently generate object hypotheses from selected salient points and emphasize a scene-centric view of segmenting RGB-D objects in a computational framework of multi-class 3D scene labeling; and (iii) we show quantitative and qualitative evaluation results on two benchmark RGB-D datasets for object detection and experimentally validate the effectiveness of the proposed method on object manipulation tasks using a mobile manipulator.

## 2. Related Work

The work presented in this paper belongs to the research scope of active segmentation or attention-driven segmentation, which was first presented by Aloimonos *et al*. [9]. It is inspired by the fact that the human visual system observes and understands a scene/image by making a series of fixations followed by segmentation. The works of Mishra *et al*. [5] have shown how segmenting a fixated object, instead of segmenting an entire scene all at once, is an easier and better defined problem. They use the concept of border ownership to find the fixation points inside the objects of interest and then extract the optimal closed contours around the points. The closed contour finally serves as the boundary of the detected object. It can be seen that active segmentation is generally comprised of two stages: (i) detecting fixation points; and (ii) segmenting object seeding from the fixation points.

Among the solutions to the problem of detecting fixation points, the most common way is to select fixations as attention points of the saliency map, which could be generated by different visual saliency detection methods. Visual saliency detection has been investigated in many disciplines, including cognitive psychology, neurobiology and computer vision. Many famous bottom-up visual attention models have been proposed [10,11]. While bottom-up attention is solely determined by the basic and low-level physical characteristics of a scene, like intensity, color, orientation, *etc*., the other stage, called top-down attention, which is influenced by tasks, emotions, expectations, *etc*., has also been suggested. Some studies [12,13,14] have also suggested that there are two schemes in saliency detection: the local and global schemes. The local scheme investigates the differences between image regions and their local surroundings, while the global scheme aims at finding salient regions that are distinct with respect to global surroundings. For the RGB-D data, a few studies have tried to investigate the effects of scene depth for saliency detection [15,16] and showed that the depth information could have a significant impact on visual attention. An interesting work presented by Potapoval *et al*. [6] is that local 3D symmetry is explored for visual saliency, and it shows that the 3D symmetry-based saliency maps capture the properties of the scene better than 2D-based saliency maps.

In this paper, we aim at finding a generic and very efficient saliency detection method that could have good detection performance for detecting salient 3D points in RGB-D scenes. Thus, we propose a new color-based saliency detection method named strength saliency (SS) that employs both local and global information for the detection. Generally, SS takes the input image as a whole system where each pixel is regarded as an entity that can exert an influence on other entities, depending on its strength and its proximity to neighboring entities. The entities interact with each other in a globally evolving process until every entity keeps an unchanged strength. The strength distribution of the whole system yields a full resolution saliency map. Thus, SS provides a new simple and efficient way to incorporate both local and global schemes into saliency detection, which supplements the state-of-the-art saliency detection methods. Besides, we employ depth cues to detect more salient points that cannot be found by the color-based method based on anisotropic center-surround difference [15].

As for the problem of segmenting hypothesized objects in an RGB-D scene, Markov random field (MRF) techniques have been previously applied to pose it as a fully-3D global multi-class segmentation problem. Lai *et al*. [1] proposed to segment objects in the reconstructed 3D scene from consecutive RGB-D images using the framework of MRF. However, the object hypotheses are not generated from the fixation points, but some pre-learned 2D object models. Therefore, it cannot detect previously unknown objects. An important technique in their method is that they apply 3D shape into MRF to achieve label smoothness and help clean up the false signals that are introduced by object hypotheses. Johnson-Roberson *et al*. [3] proposed to create color models for hypothesized objects from seed points and to perform multi-class segmentation. The segmentation procedure is iterated, while the color models are updated. In this work, we propose to generate object hypotheses from seed points using the 3D shape (especially object size) and find that this simple and quick method is sufficient when seeding from the fixation points detected by our saliency method. We combine cues from object hypotheses and 3D shape for MRF and employ color cues to further refine the labeling results. Thus, our method utilizes both 3D shape and color information to seek more robustness and accuracy in object detection.

## 3. Detection of Unknown RGB-D Objects

To address the problem of unknown object detection in RGB-D images, we emphasize a scene-centric view where an RGB-D image is deemed to be one part of an independent 3D scene. We then represent the part of a 3D scene as a set of voxels *V*. Each voxel *v* is associated with a label $y_{v}\in \left\{1,\cdots ,C,c_{B}\right\}$, where 1,⋯,C are hypothesized object classes and $c_{B}$ is the background class. Therefore, the problem of unknown object detection could be considered as a multi-class labeling problem in 3D scene. The main challenge in our scenario is that the set of object classes is previously unknown and needs to be generated online by multi-object hypotheses. At the same time, the detection method also needs to deal with the inaccuracy and uncertainty of object hypotheses due to unavailable prior information. We then model the joint distribution of voxel class labels using MRF-based techniques, which have been used for many labeling tasks and can provide a unified computational framework for combining local evidence with dependencies across regions. The optimal labeling of the 3D scene minimizes the following energy:

(1) $$E\left(y_{1},\cdots ,y_{\left|V\right|}\right)=\sum_{v\in V}\varphi_{v}\left(y_{v}\right)+\sum_{\{i,j\}\in N}\phi_{i,j}\left(y_{i},y_{j}\right)$$

where *N* is the set of all pairs of neighboring voxels. The data term $\varphi_{v}\left(y_{v}\right)$ measures how well the assigned label fits the observed data, and the pairwise term $\phi_{i,j}\left(y_{i},y_{j}\right)$ models interactions between adjacent voxels, like label smoothness. The data term in MRF is typically represented as the negative log likelihood:

(2) $$\varphi_{v}\left(y_{v}\right)=-\ln p\left(y_{v}|\Omega_{v}\right)=-\frac{1}{|\Omega_{v}|}\sum_{x\in \Omega_{v}}\ln p\left(y_{v}|x\right)$$

where *x* denotes a 3D point, $\Omega_{v}$ is the set of 3D points in the voxel *v* and $p(y_{v}|x)$ is the probability of point *x* belonging to class $y_{v}$. We will generate multi-object hypotheses (see Section 3.2) based on saliency detection (see Section 3.1). The set of possible object classes is obtained, and the probability of each point belonging to an object class is further modeled. The pairwise term will be discussed in Section 3.3, where we also introduce an intuitive method to refine the detection results.

### 3.1. Generation of the Visual Saliency Map

In this work, we explore how to rapidly detect the possible positions of unknown objects in a biologically-inspired way. A novel saliency detection method, named strength saliency (SS), is proposed to generate saliency maps that can be further used for predicting human fixation, so as to obtain the possible object positions. The underlying idea is that a given image is treated as a system, where each pixel can exert an influence on other pixels, depending on its strength and its proximity to neighboring pixels. In our implementation, we build on the premise that image borders are not salient or uninteresting, and hence, their pixels act as initial entities with high strength. The more similar in feature space an entity is to another, the more similar their strengths will be. The entities interact with each other until every entity keeps an unchanged strength. The strength distribution of the whole system yields the full resolution saliency map.

Specifically, we detect image saliency in the CIE L*a*b* (CIELAB) color space; thus, only the color image is used. An input RGB image *I* is represented in terms of pixels *P* with specific features *F*, $I=\{\left(P_{i},F_{i}\right)\},i=1,\cdots ,M$, where *M* is the total number of pixels. $S(P_{i})$ is denoted as the strength of $P_{i}$. All pixels located at the four image borders are selected as the initial entities with a strength of one. The width or height of image border is defined as *δ*. The proximity between entities is defined as the spatial distance at multi-scale *s* as the 8s neighborhood, as well as the appearance distance in the corresponding feature space. We set s=1,⋯,4 in the experiments. The induced interaction means between entities is defined as:

(3) $$U\left(P_{i},P_{o}\right)=\eta \left(P_{o}\right)\left[1-\frac{d(P_{i},P_{o})}{MaxDist}\right]$$

where $P_{i}$ is one of the 8s neighboring pixels of $P_{o}$, η denotes the gain with $\eta \left(P_{o}\right)=S\left(P_{o}\right)$, $d(P_{i},P_{o})$ represents the L2 distance between $P_{i}$ and $P_{o}$ in the selected feature space and MaxDist is the maximum distance between any two feature vectors in the feature space. We select the feature space as the separated CIELAB color channel. Equation (3) shows that the more similar in feature space a pixel is to another, the more similar their strengths will be. Since an entity will obtain various influences from different sources, we update the strength of $P_{i}$ by:

(4) $$S\left(P_{i}\right)=\max\left\{U\left(P_{i},P_{j}\right)\right\},j=1,\cdots ,N_{i}$$

where $N_{i}$ is the number of entities that will affect $P_{i}$, and its strength is selected as the maximum value generated from different sources. Based on Equations (3) and (4), the updating procedure can be iterated until each pixel keeps an unchanged strength. The saliency of any pixel $P_{i}$ is finally calculated as $1-S(P_{i})$, which yields a full-field saliency map. 

**Algorithm 1** Algorithm of SS

**Figure 2:**
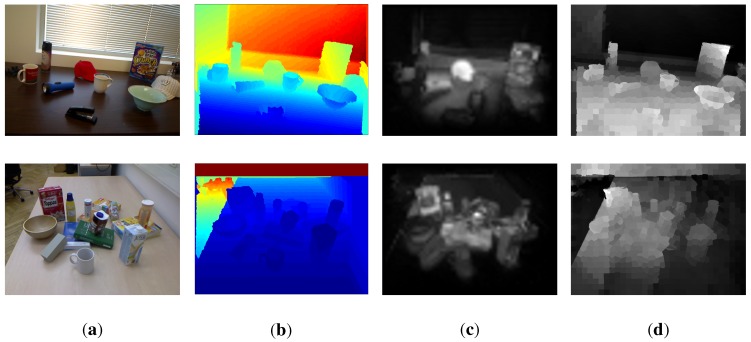
Given a pair of an RGB image (**a**) and a depth image (**b**), the proposed algorithm strength saliency (SS) outputs the color-based saliency map (**c**), while the employed method [15] outputs the depth-based saliency map (**d**). The two types of saliency maps supplement each other to find sufficient salient positions.

**Input:** An input image *I* with height *H* and width *W*
**Output:** The final saliency map *S*
  1: *sumSal* = *Zeros*(*H*,*W*); *k* = 0;
  2: **for** *scale* = 1 to *s* **do**
  3:   **for** each CIELAB color channel **do**
  4:     *k* = *k* + 1
  5:     Set width or height of image border as *δ* = *scale*;   
  6:     Associate all pixels in the four borders with a strength of one;
  7:     Iterate using Equations (3) and (4) until each pixel *P_i_* keeps an unchanged strength *S*(*P_i_*);
  8:     Get a full-field saliency map *salMap*[*k*], each pixel *P_i_* of which is assigned with the saliency value 1 − *S*(*P_i_*);
  9:     *sumSal* = *sumSal* + *salMap*[*k*];
  10:  **end for**
  11: **end for**
  12: *S* = *L2Normalization*(*sumSal*);


Base on the above framework, we could generate a number of full-field saliency maps at different neighborhood scales along with different feature spaces. These maps are summed and normalized using L2-normalization. The overall algorithm is summarized in Algorithm 1. Some example results are shown in the third column of Figure 2. We will evaluate the proposed algorithm on several benchmark datasets and show its good performance in Section 4.1. Although color cues play important roles in saliency detection, they cannot detect those objects that have a similar color appearance with the background. For example, the stapler in the scene cannot be highlighted from its surroundings (see Figure 2). Therefore, we employ a depth saliency method [15] based on the anisotropic center-surround difference to enhance our color-based saliency detection method in order to find more salient positions. This measures how much a point stands out from its surroundings based on the depth values of these points, while taking the global depth structure into consideration. The fourth column of Figure 2 shows the depth-based saliency maps. The color-based saliency map and depth-based saliency map are then fed into the saliency seeding procedure, which is introduced in the next subsection.

### 3.2. Saliency Seeding and Modeling Hypothesized Objects

To estimate the possible object positions from the output saliency map, the simplest way would be using a fixed threshold. However, this method would become unfavorable when the peaks show large variance among salient regions. The opening-by-reconstruction operation followed by finding the regional maxima are performed to retrieve rough object regions [17]. Specifically, the morphological erosion is performed on the saliency map using a 20-pixel disk-shaped structuring element to flatten undesirable small peaks while keeping desirable large peaks. The morphological reconstruction is then performed on the eroded image using the original image as a mask. Regional maxima are then computed, and flat peaks are selected as rough object regions. The centroids of these regions are then selected as candidate seed points of the hypothesized objects. We further remove the seed points, which lie on the dominating 3D support surface that is perpendicular to the gravity vector. Specifically, we first calculate the gravity vector using a simple yet robust method [18]. This method tries to find the direction that is the most aligned to or most orthogonal to locally estimated surface normal directions at as many points as possible. The points that have consistent normal vectors with the gravity vector are then selected as support surface points. The dominating support surface is finally extracted, such that most of the selected points have almost the same height in the world coordinate frame. Figure 1a shows the generated seed points for an example image.

**Figure 3:**
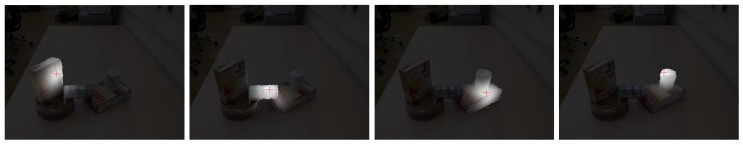
Examples of modeling hypothesized objects from 4 seed points (denoted by the red plus), respectively, to obtain probability maps defined on all pixels.

Suppose that *C* seed points have been generated; the set of hypothesized object classes is formed as {1,⋯,C}. We propose to model an object hypothesis by using the 3D shape:

(5) $$p\left(c|x\right)=\exp\left(-\gamma ∥x-x_{0}{∥}^{2}\right),x\in PC,c\in \left\{1,\cdots ,C\right\}$$

where $x_{0}$ is the 3D point of the corresponding seed point, *x* denotes any point in the point cloud PC of the input image and γ controls how quickly the probability decreases with increasing spatial distance in meters. The larger γ is, the smaller the 3D size of the object hypothesis is. Figure 3 shows some generated object hypotheses from different seed points when γ=180. In the experiments, we will investigate how γ can affect the accuracy of object detection. It can also be seen from Figure 3 that our model may only obtain a probability map of one part of a true object. Even though, it shows that different parts of a true object with different labels can be smoothed by the MRF to a certain degree. Especially, they could be further merged by an intuitive refinement method, which will be introduced in the next subsection. To obtain a background probability, we take advantage of the foreground probabilities of *C* object hypotheses:

(6) $$p\left(c_{B}|x\right)=\alpha \min_{c\in [1,C]}\left\{1-p\left(c|x\right)\right\}$$

where α is empirically set to 0.1. Additionally, the points that lie on the dominating support surface are associated with a probability value of one. By using Equations (5) and (6), the data term in Equation (2) can be easily calculated.

### 3.3. Extraction of the RGB-D Objects

After modeling the data term in MRF, as shown in Equation (1), we would like to further enforce contextual constraints on the scene labeling. For example, voxels on a smooth surface should be assigned the same label. We employ Lai’s model [1] to encode interactions between nearby voxels:

(7) $$\phi_{i,j}\left(y_{i},y_{j}\right)=\lambda \frac{1_{y_{i}\neq y_{j}}}{d(n_{i},n_{j})}\left(I\left(n_{i},n_{j}\right)+\varepsilon \right)$$

where λ and ε are balancing parameters, $1_{y_{i}\neq y_{j}}$ evaluates to one when $y_{i}\neq y_{j}$, $d(n_{i},n_{j})$ measures the difference between surface normal $n_{i}$ and $n_{j}$ of, respectively, voxels *i* and *j*. Specifically, the L2-distance plus a small constant is used as the distance metric between surface normals. $I(n_{i},n_{j})$ indicates whether the surface transition between voxels *i* and *j* is convex, which is calculated by:

(8) $$I\left(n_{i},n_{j}\right)=\left[\left(n_{i}-n_{j}\right)\cdot \left(i-j\right)>0\right]$$

It can be seen from Equation (7) that those pairs of voxels with similar normals and convex surface transitions will cost much if they are assigned different class labels. This pairwise term and the data term together define a multi-class pairwise MRF, as shown in Equation (1), whose energy could be quickly minimized using graph cuts [19].

**Figure 4:**
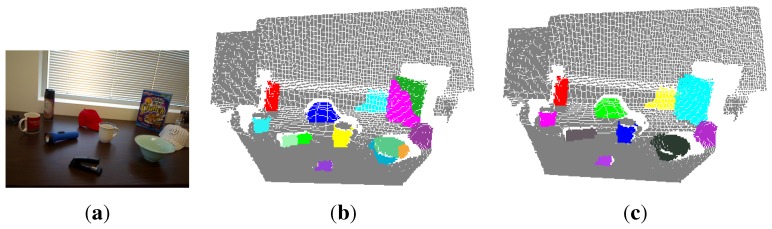
The multi-class labeling result (**b**) for an example input RGB-D image (**a**) (only the RGB image is shown here) after performing the Markov random field (MRF) optimization; (**c**) the final result after performing refinement operations. Different objects are randomly colored.

Figure 4 shows the multi-class labeling result for an example input RGB-D image after performing the MRF optimization. We can see that 14 objects are segmented out from the scene, even if more than 14 hypothesized objects are modeled. This means that the MRF could deal with the inaccuracies and uncertainties of object hypotheses to a certain extent. However, the objects, such as the flashlight, bowl and cereal box, are not labeled as one object, respectively. To address this problem, we propose to further refine the labeling results in an intuitive way, such that the spatially-connected point clouds with consistent color models should be merged into one object. For example, the flashlight is labeled as two objects. The corresponding two point clouds are spatially connected and have a similar color appearance. We model the object appearance using a 3D color histogram. Each RGB color channel is split into 20 bins, thus making 8000 bins in total for representing the color histogram on which L2 normalization is performed. We further calculate the correlation coefficient to measure the similarity between two color histograms. If the correlation coefficient is greater than $\tau_{ce}$, which is empirically set to 0.5, we deem that the two connected objects should be two parts of a true object, and the corresponding point clouds should be merged and labeled with one class. By performing these operations, the multi-class labeling result shown in Figure 4b is refined and shown in Figure 4c. We can see that the wrongly-segmented parts of true objects are correctly merged, which makes the actively-detected objects more consistent with humans’ perception and segmentation.

## 4. Results and Discussion

To validate the performance of the proposed unknown object detection method, especially for the common robotic manipulation tasks, we use two publicly available RGB-D datasets, object segmentation dataset (OSD) [7] and RGB-D scene dataset [20], captured by a Kinect-style sensor to evaluate how successfully the proposed method detects unknown objects. Both datasets incorporate sorts of common indoor scenes where the daily objects are randomly placed on the counter tops, tables, desks, grounds, *etc*. In some scenes, the objects are stacked and occluded, making object detection much more challenging. In addition, since our task is focused on object manipulation, the objects we study have a small and graspable size. Therefore, we mainly focus on detecting the unknown daily objects in the evaluations and experiments.

The ground truth of objects is represented as the bounding boxes around the objects of interest. Therefore, we project the point clouds of detected objects in a 3D scene into 2D image and calculate the overlap between the bounding box of projected pixels and the ground truth bounding box. The overlap is computed by the ratio between the intersection and union of the bounding boxes. If the overlap is greater than 0.5, the object is considered detected. The precision, recall and F1-measure scores are calculated to demonstrate the performance of our method. Since the proposed saliency detection method plays an important role in the whole object detection process, we first carry out human fixation prediction experiments to quantitatively and qualitatively evaluate the proposed saliency detection method on several benchmark datasets. Second, we report the object detection accuracy on the two benchmark RGB-D datasets. Finally, the proposed method is applied to detect unknown objects in the real robotic scenes. The detected objects are then manipulated by a mobile manipulator when it is asked to perform actions, such as cleaning up the ground.

### 4.1. Evaluation of Saliency Detection

We perform human fixation prediction experiments to evaluate the proposed saliency detection method. Three eye movement datasets, MIT [21], Toronto [22] and Kootstra [23], which are publicly available, are used as benchmark datasets. The first dataset, MIT [21], introduced by Judd *et al*., contains 1003 landscape and portrait image. The second dataset Toronto [22], introduced by Bruce *et al*., contains 120 images from indoor and outdoor scenes. The Kootstra [23] dataset contains 100 images, including animals, flowers, cars and other natural scenes. In order to quantitatively evaluate the consistency between a particular saliency map and a set of eye-tracked fixations of the image, we use three metrics: ROC area under the curve (AUC), normalized scanpath saliency (NSS) and the correlation coefficient (CC). For the AUC metric, we use a type of implementation, AUC-Borji [24]. These metric codes are available on the website [25]. We compare the proposed method SS with eight state-of-the-art saliency detection methods, including Itti2 [26], SigSal [27], GBVS [26], SUN [28], AIM [22], LP [21], CAS [12] and BMS [14]. For the sake of simplicity, the compared methods are named with no extra meaning here.

**Figure 5:**
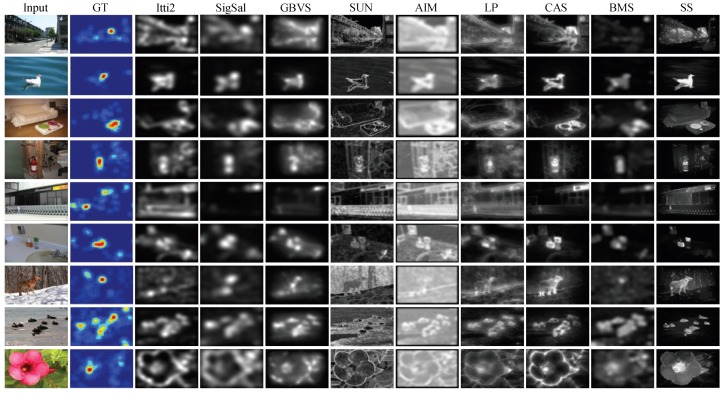
Comparison of saliency maps from nine methods on three benchmark eye movement datasets. The first two columns are the sample images and their fixation heat maps from the MIT, Toronto and Kootstra datasets (each dataset shows herein three sample images). The fixation heat maps are computed by applying Gaussian blur on the raw eye fixation maps. The rest of the columns show the saliency maps from the state-of-the-art methods and our method, SS.

The evaluation metrics are quite sensitive to blurring. Parameterized by the Gaussian blur standard deviation (STD) in image width, the factor is explicitly analyzed to provide a better understanding of the comparative performance of each method. We set Gaussian blur STD from 0–0.16 in image width. The optimal AUC-Borji, NSS and CC scores of each method together with the corresponding Gaussian blur STD on the three benchmark datasets are reported in Table 1, Table 2 and Table 3. For each metric, the proposed method SS achieves top performance when comparing the average scores in each method on all three benchmark datasets. The methods GBVS and BMS are also competitive among these compared methods. Figure 5 shows sample saliency maps generated by SS and the other eight state-of-the-art methods. The sample input images are randomly selected from the three benchmark datasets. From this figure, we can see that many of the compared methods tend to favor the boundaries, rather than the interior region of salient objects. Comparably, our method SS can perform much better in terms of detecting not only the salient boundaries, but also interior regions. Such advantages can facilitate the detection of unknown objects in the later process.

**Table 1:** Average AUC-Borji score with optimal blurring. The highest score on each dataset is shown in bold. The second and third highest are underlined.

Dataset	Itti2 [26]	SigSal [27]	GBVS [26]	SUN [28]	AIM [22]	LP [21]	CAS [12]	BMS [14]	SS
MIT [21]	0.7909	0.7678	0.8236	0.7128	0.8095	0.7703	0.7610	0.7868	**0.8299**
Optimal STD	0.16	0.16	0.16	0.07	0.16	0.14	0.10	0.11	0.08
Toronto [22]	0.8071	0.7921	0.8248	0.7069	0.7970	0.7854	0.7791	0.7960	**0.8270**
Optimal STD	0.14	0.12	0.10	0.05	0.16	0.12	0.08	0.08	0.07
Kootstra [23]	0.6467	0.6528	0.6674	0.5699	0.6622	0.6429	0.6445	0.6655	**0.6789**
Optimal STD	0.10	0.11	0.05	0.05	0.08	0.09	0.06	0.05	0.06
Average	0.7482	0.7376	0.7720	0.6632	0.7563	0.7329	0.7282	0.7495	**0.7786**

**Table 2:** Average normalized scanpath saliency (NSS) score with optimal blurring. The highest score on each dataset is shown in bold. The second and third highest are underlined.

Dataset	Itti2 [26]	SigSal [27]	GBVS [26]	SUN [28]	AIM [22]	LP [21]	CAS [12]	BMS [14]	SS
MIT [21]	1.1542	1.1083	**1.3821**	0.8677	1.0355	1.0478	1.1021	1.2627	1.3817
Optimal STD	0.11	0.06	0.01	0.05	0.16	0.05	0.05	0.05	0.05
Toronto [22]	1.3083	1.3787	**1.5194**	0.8120	1.0015	1.1640	1.2878	1.5191	1.4530
Optimal STD	0.05	0.00	0.00	0.04	0.16	0.02	0.03	0.00	0.04
Kootstra [23]	0.5415	0.5693	0.6318	0.2829	0.5411	0.5363	0.5587	**0.7014**	0.6968
Optimal STD	0.08	0.07	0.02	0.04	0.10	0.06	0.04	0.04	0.04
Average	1.0013	1.0188	**1.1778**	0.6542	0.8593	0.9160	0.9828	1.1610	1.1772

**Table 3:** Average CC score with optimal blurring. The highest score on each dataset is shown in bold. The second and third highest are underlined.

Dataset	Itti2 [26]	SigSal [27]	GBVS [26]	SUN [28]	AIM [22]	LP [21]	CAS [12]	BMS [14]	SS
MIT [21]	0.1855	0.1766	0.2211	0.1388	0.1691	0.1670	0.1750	0.2004	**0.2229**
Optimal STD	0.11	0.06	0.01	0.05	0.16	0.05	0.05	0.05	0.06
Toronto [22]	0.3941	0.4050	**0.4551**	0.2398	0.3133	0.3469	0.3770	0.4401	0.4401
Optimal STD	0.07	0.00	0.00	0.04	0.16	0.04	0.03	0.03	0.05
Kootstra [23]	0.2652	0.2741	0.3088	0.1310	0.2716	0.2568	0.2633	0.3234	**0.3365**
Optimal STD	0.08	0.08	0.03	0.04	0.10	0.07	0.05	0.05	0.04
Average	0.2816	0.2852	0.3283	0.1699	0.2513	0.2569	0.2718	0.3213	**0.3332**

### 4.2. Evaluation of Unknown Object Detection

**Figure 6:**
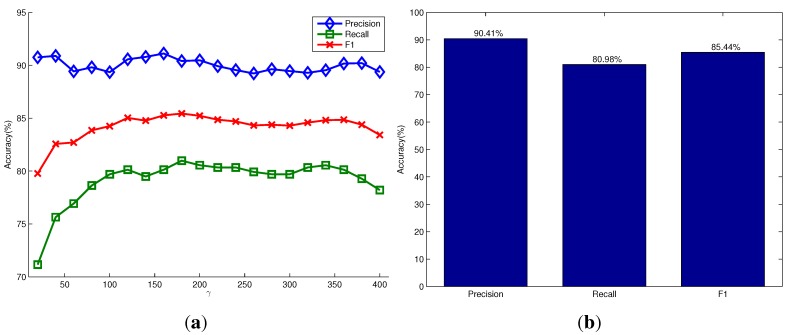
Quantitative evaluation on the OSD dataset. (**a**) The precision, recall and F1-measure scores *versus* the parameter γ in the model of the object hypothesis, respectively; (**b**) the corresponding scores when using γ=180.

**Figure 7:**
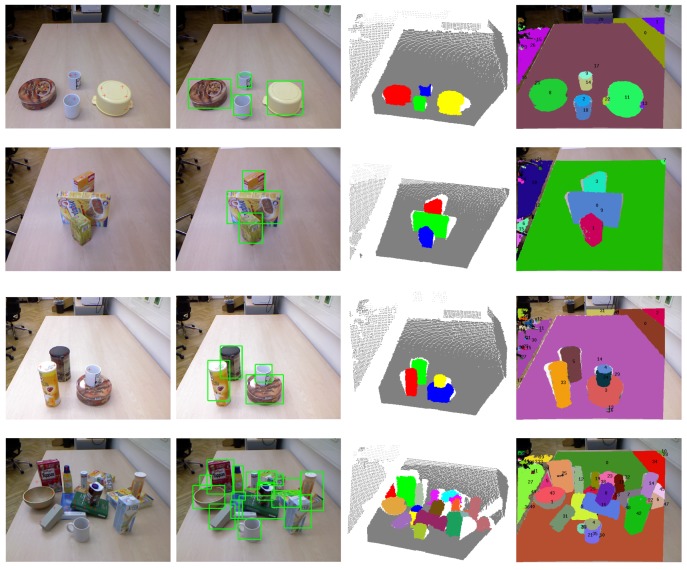
Visual examples of unknown object detection on the OSD dataset. The first column shows the input scene images overlapped with the detected seed points (red plus) of object hypotheses. The second and third columns show the bounding boxes and colored point clouds of detected objects, respectively. The last column shows the detection results using the method proposed in [7].

In the OSD dataset [7], there are 111 RGB-D images of objects on a table. This dataset is very challenging because different types of daily objects are randomly located, stacked and occluded. We detect unknown objects for each pair of RGB and depth images and report the precision, recall and F1-measure scores of object detection over the whole dataset. The detection results are shown in Figure 6. Figure 6a shows the variations of the three types of detection accuracy under different settings of the parameter γ in the model of the object hypotheses. It shows that the parameter γ has little influence on the detection accuracy in this dataset. Our method can achieve a high detection precision of more than 90% due to the advantage that the proposed saliency detection and seeding methods could generate more seed points of salient objects in the foreground than the non-salient objects in the background. The recall accuracy of detection is relatively low, but still achieves about 80%. This is because most images in this dataset contain objects that are stacked and occluded. Different parts of the occluded objects are always segmented as different objects, while the stacked objects with a similar appearance are segmented as one object. Figure 6b shows the corresponding scores when using the relatively optimal parameter γ=180, and it achieves the highest F1-measure score. Figure 7 shows some qualitative results of our method, as well as the method proposed by Richtsfeld *et al*. [7]. It shows that the proposed saliency-guided detection method can remove many irrelevant backgrounds and produce a smaller number of object proposals than the method in [7], which segments objects in the whole image. Although the method in [7] aims at clustering object surface patches in the whole image using machine learning, there exists an over-segmentation problem. For example, there are only four objects of interest in the first scene of Figure 7, but the method in [7] outputs almost 26 objects. The characteristics of these methods will be compared next.

**Figure 8:**
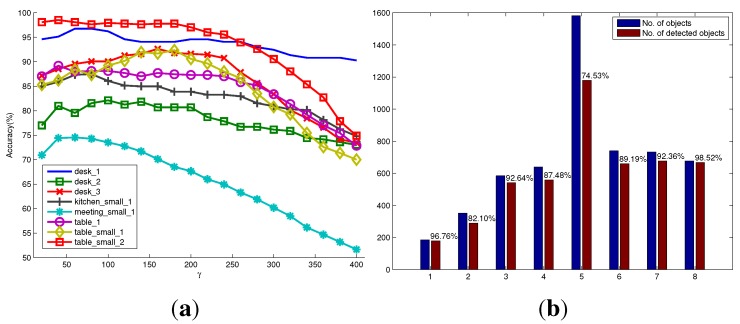
Quantitative evaluation on the RGB-D scene dataset with eight scenarios. (**a**) The successful detection percentages *versus* the parameter γ in the model of object hypothesis for eight scenarios, respectively; (**b**) the number of objects, as well as the number of detected objects in each scenario using the optimal parameters.

**Figure 9:**
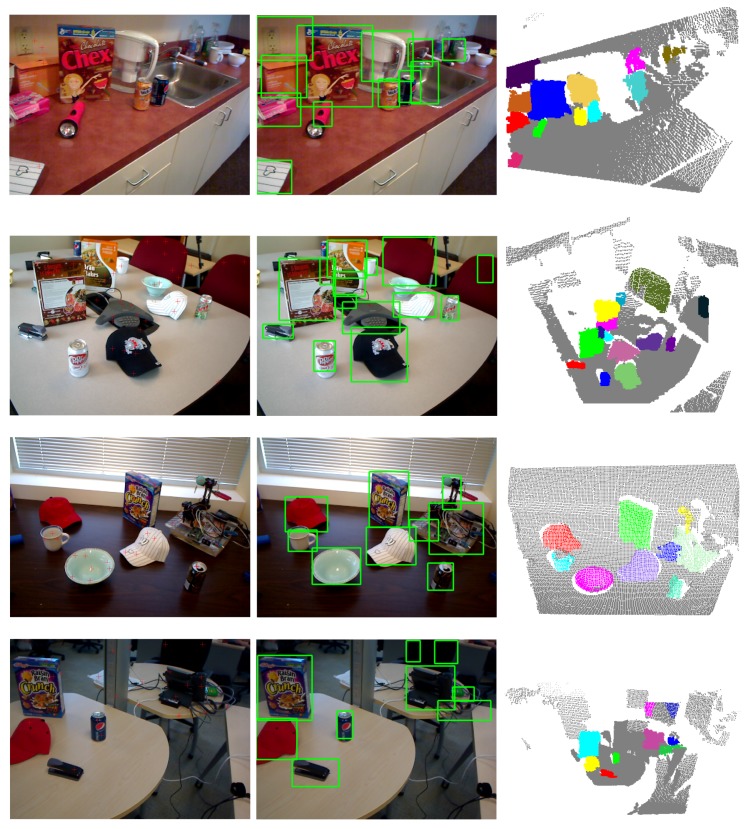
Visual examples of unknown object detection on the RGB-D scene dataset.

In the RGB-D scene dataset [20], there are six categories of objects (e.g., bow, cap, cereal box, coffee mug, flashlight and soda can), which appear in different scene settings, such as in a kitchen, in a meeting room, on a desk and on a table. These settings result in a total of eight scenarios in the dataset. Each scenario contains a sequence of RGB-D images taken from different views. The success rate of object detection only for these six categories of objects are reported, since the ground truth of other categories of objects are not available. The detection results are shown in Figure 8. Figure 8a shows the variation of detection accuracy (recall accuracy) on each scenario under different settings of the parameter γ. For most scenarios in the RGB-D scene dataset, the optimal or suboptimal γ for achieving a high recall accuracy of object detection is around 80. This means that we could generate object hypotheses with a little bit larger size, so as to reduce the possibility of over-segmenting. However, it will inevitably sacrifice the detection precision. Empirically, to deal with unpredictable scenes when considering precision and recall accuracy, the good performance of unknown object detection could be achieved by assigning γ with 180. In addition, we found that the parameter γ is related to the method of how to generate seed points of object hypotheses. Other methods of generating seed points may have different optimal γ from what is reported in this paper. Figure 8b shows the number of successfully detected objects compared to the total number of objects in each scenario when using the optimal parameters. It can be seen that in the eight scenario table_small_2, a high detection accuracy up to 98.52% is achieved. This is mainly because this scenario contains only four foreground objects that have almost different appearances, and the background is simple. However, the fifth scenario meeting_small_1 is more challenging. The number of objects in each image is always more than 10. They are often occluded by other objects or the image borders. Especially, many salient objects are not labeled as foreground objects in the ground truth. Thus, the detection success rate is relatively low. In general, the detection rates over the whole dataset are satisfactory and validate the good performance of the proposed method. As shown in Table 4, our results are competitive with the detection rates reported in [5], even though a larger number of images for each scenario in the dataset is used for detection in our evaluation. Some qualitative results are also shown in Figure 9.

**Table 4:** Comparison of the detection rate on the RGB-D scene dataset.

Scenario	Mishra *et al.* [5]		Ours	
	No. of Objects	% of Objects Detected	No. of Objects	% of Objects Detected
desk_1	162	94.4%	185	96.8%
desk_2	301	94.0%	352	82.1%
desk_3	472	96.0%	584	92.6%
kitchen_small_1	502	82.3%	639	87.5%
meeting_small_1	1047	83.0%	1582	74.5%
table_1	554	92.8%	740	89.2%
table_small_1	666	90.7%	733	92.4%
table_small_2	584	97.6%	677	98.5%

Table 5 reports the running time of our current single-threaded C++ implementation of the proposed method for a typical 640×480 indoor scene RGB-D image. It runs on a 2.4-GHz dual-core 64-bit Linux laptop with 16 GB of memory. In the first stage of active segmentation, it takes about 0.23 s to detect fixation points using color and depth cues. The overwhelming majority of computation is spent on the stage of segmentation, where the calculation of 3D point normals and MRF optimization are time-consuming. Object hypothesis generation, refinement of labeled objects and rendering of detected objects take negligible time. Overall, it requires around 3 s to process an RGB-D frame using our current computing hardware.

**Table 5:** Running time of the proposed method.

Detection of Fixation Points	Segmentation	Overall
0.23 s	2.7 s	2.93 s

Table 6 compares the methods in terms of some characteristics. Different from the other two methods that are both biologically inspired, the method in [7] does not follow the scheme of active segmentation and aims at clustering object surface patches in the whole image using machine learning. Therefore, the segmentation performance would depend on the training set, and it would produce a larger number of object proposals. The method in [5] relies highly on the edge detector in [29], which is shown to be very time consuming. It also needs training to learn some parameters for determining the depth boundary in the method. Generally, the proposed method is shown to be more generic and efficient.

**Table 6:** Comparison of the method characteristics.

Method	Method Characteristics			
	Biologically Inspired	Need Training	Rely on Edge Detection	Computational Efficiency
Richtsfeld *et al*. [7]	No	Yes	No	Medium (2–5 s)
Mishra *et al*. [5]	Yes	Partially	Yes	Low (>5 s)
Ours	Yes	No	No	Medium (2–5 s)

### 4.3. Unknown Object Detection and Manipulation

**Figure 10:**
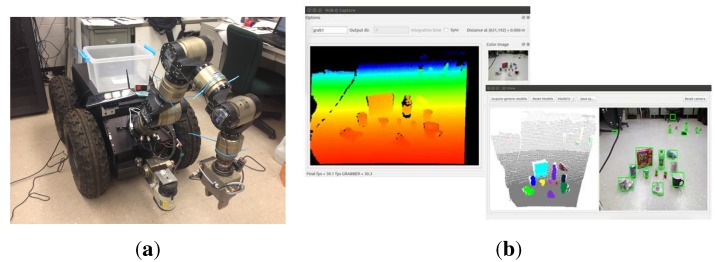
Our mobile manipulator (**a**) and the developed GUI (**b**) for observing the object detection process.

We also test our object detection method for manipulation tasks using a mobile manipulator system equipped with a Kinect camera, as shown in Figure 10a. This mobile manipulator consists of a seven DOF manipulator and a nonholonomic mobile base. We implemented our object detection algorithm in the perception module of the robot platform based on the core library Nestk developed in [30]. The developed GUI for observing the object detection process is shown in Figure 10b. Currently, it takes our version of implementation about three seconds to process a typical indoor scene. In the experiment, the mobile manipulator detects unknown objects using the perception module and calculates the attributes (*i.e*., object size, grasping position, *etc*.) of the detected objects. When the robot is commanded to perform a task like “cleaning up the ground” via the dialogue-based interface, the robot starts to process the human utterance through the natural language processing module, the grounding module and the action module, which have been developed in our previous works [31]. The task is then converted to a sequence of robot actions (*i.e*., move to, open gripper, close gripper, *etc*.) and trajectories for the mobile manipulator to execute.

**Figure 11:**
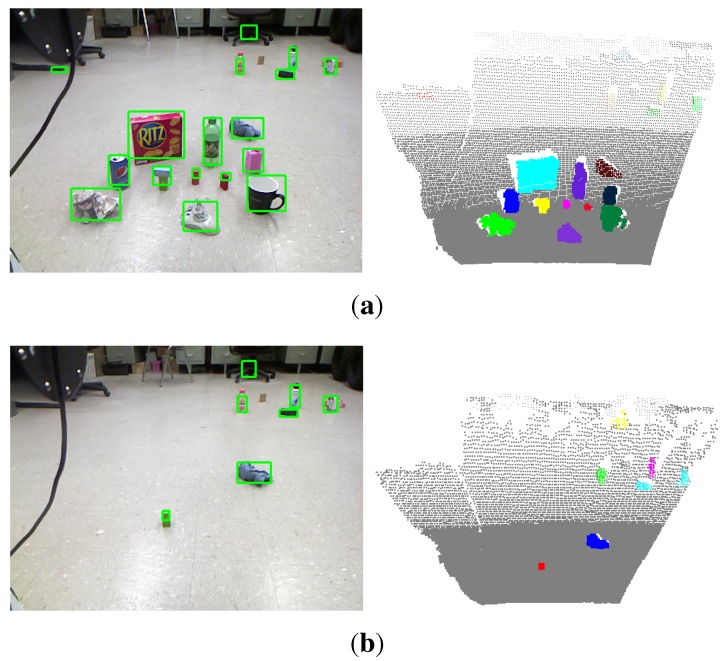
A typical experimental scene seen from the robot. (**a**,**b**) show the detected objects before and after the robot picking up all detected objects within its manipulation range, respectively.

**Figure 12:**
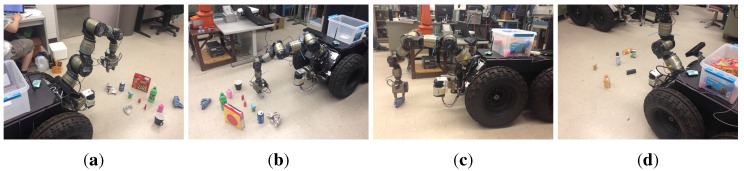
Snapshots of scenes when our robot is performing the task of cleaning up the ground. After detecting the unknown objects, the robot moves its gripper to the nearest object (**a**), grasps the object (**b**) and then puts it into the box. When the objects are out reach of the manipulator, the robot moves its base to facilitate grasping (**c**). After recycling all objects in the current spot, the robot continues to find objects and moves to the next spot (**d**).

A typical experimental scene is shown in Figure 11. Several daily used objects are randomly placed on the ground of our laboratory. The robot is commanded to automatically find, pick up these objects and put them into the box on the top of the robot base. At the beginning, the robot detects the objects in its field of view, as shown in Figure 11a, and starts to pick up the objects within its manipulation range. After a round of manipulations, the scene shows that the green block and the blue cleaning rag have not been recycled yet, as shown in Figure 11b. This is because the green block is stacked with another block and has not been detected before, and the blue cleaning rag is out reach of the manipulator. Thus, the robot detects the objects in the current scene again to make sure that there is no object left before navigating to the next spot. After detecting and picking up the green block, the robot moves to the cleaning rag and then picks it up. The robot repeats the procedures of detecting objects, approaching the objects, detecting objects again and picking them up. Figure 12 shows some typical snapshots of scenes when the robot is performing these procedures automatically.

## 5. Conclusions

We presented an unknown object detection approach that utilizes saliency detection and 3D multi-class labeling techniques. The approach generates object hypotheses from the detected salient 3D points using the 3D shape and performs multi-class labeling on an MRF over the voxels of the 3D scene. In addition, object detection is performed on a part of 3D scene, because of using only one RGB-D image for each time of detection, but we believe that the scene-centric paradigm allows for segmenting multiple objects from multiple views that could be used to reconstruct a whole scene. We have validated the proposed saliency detection method on three eye movement datasets and shown object detection results on two benchmark RGB-D datasets. We also have applied the proposed approach to our mobile manipulator to execute the tasks, such as cleaning up the ground.

Detection of unknown objects is a very promising area of research in robotics, specifically due to the growing demand of dealing with new objects in new environments. Future direction could explore semantic attributes, like part, shape and material, to help describe the object hypotheses seeding from the salient points. Through describing objects using semantic attributes, the object hypotheses can be well refined, and more information about the detected objects are provided. Besides, providing sufficient information about the detected objects can also benefit the referential grounding based on which, humans could interact with robots through natural language to achieve a common goal.
